# Identification of Novel Inhibitors against Coactivator Associated Arginine Methyltransferase 1 Based on Virtual Screening and Biological Assays

**DOI:** 10.1155/2016/7086390

**Published:** 2016-10-27

**Authors:** Fei Ye, Weiyao Zhang, Wenchao Lu, Yiqian Xie, Hao Jiang, Jia Jin, Cheng Luo

**Affiliations:** ^1^College of Life Sciences, Zhejiang Sci-Tech University, Hangzhou, China; ^2^Key Laboratory of Plant Secondary Metabolism and Regulation of Zhejiang Province, Hangzhou, China; ^3^Drug Discovery and Design Center, State Key Laboratory of Drug Research, Shanghai Institute of Materia Medica, Chinese Academy of Sciences, Shanghai, China; ^4^University of Chinese Academy of Sciences, Beijing, China

## Abstract

Overexpression of coactivator associated arginine methyltransferase 1 (CARM1), a protein arginine N-methyltransferase (PRMT) family enzyme, is associated with various diseases including cancers. Consequently, the development of small-molecule inhibitors targeting PRMTs has significant value for both research and therapeutic purposes. In this study, together with structure-based virtual screening with biochemical assays, two compounds DC_C11 and DC_C66 were identified as novel inhibitors of CARM1. Cellular studies revealed that the two inhibitors are cell membrane permeable and effectively blocked proliferation of cancer cells including HELA, K562, and MCF7. We further predicted the binding mode of these inhibitors through molecular docking analysis, which indicated that the inhibitors competitively occupied the binding site of the substrate and destroyed the protein-protein interactions between CARM1 and its substrates. Overall, this study has shed light on the development of small-molecule CARM1 inhibitors with novel scaffolds.

## 1. Introduction

Arginine methylation is an important posttranslational modification catalyzed by protein arginine N-methyltransferases (PRMTs) [1, 2]. During PRMT catalysis, the methyl group of S-adenosyl-L-methionine (AdoMet, SAM) is transferred to the guanidino group of the target arginine, resulting in mono- or dimethylated arginine residues along with S-adenosyl-L-homocysteine (AdoHcy, SAH) as a coproduct [3]. There are nine PRMTs identified so far, which can be classified into three categories: type I (PRMT1, 2, 3, 4, 6, and 8), type II (PRMT5 and 9) and type III (PRM7) [4]. Type I PRMTs catalyze mono- and asymmetric dimethylation of arginine residues, whereas type II PRMTs catalyze mono- and symmetric dimethylation of arginine residues [5]. PRMT7 is the only known type III PRMT, which catalyzes monomethylation of arginine [6].

PRMT4, also known as CARM1 (coactivator associated arginine methyltransferase 1) methylates a wide variety of histone and nonhistone substrates including H3R17, H3R26 [7], SRC-3 [8], CBP/p300 [9], NCOA2 [10], PABP1 [11], and SmB [12]. Consequently, CARM1 participates in many cellular processes by impacting chromatin architecture and transcriptional initiation [9, 13], RNA processing and stability [14], and RNA splicing [12]. Overexpression of CARM1 has been observed in multiple cancer types including myelocytic leukemia [15] and breast [10], prostate [16], lung [17], and colorectal carcinomas [18], making it a potential target for anticancer therapy.

Due to essential roles of CARM1 in the regulation of cellular functions as well as tumorigenesis, discovery of CARM1 inhibitors has recently attracted much attention. To date, a number of CARM1 inhibitors have been reported [19–27] (see Figure S1 in Supplementary Material available online at http://dx.doi.org/10.1155/2016/7086390). According to the chemical structures, these inhibitors can be divided into several categories: (i) 3,5-bis(bromohydroxybenzylidene) piperidin-4-one inhibitors (compounds 1-2 in Figure S1), (ii) pyrazole inhibitors (compounds 3–10 in Figure S1), (iii) benzo[*d*]imidazole inhibitors (compounds 11–13 in Figure S1), and (iv) other inhibitors (compounds 14-15 in Figure S1) [28]. However, the majority of these inhibitors are lacking selectivity and drug-likeness; thus turning these inhibitors into therapeutically useful compounds is challenging. Therefore, it is still of significant interest to discover selective inhibitors targeting CARM1 with good pharmacological properties.

Virtual screening is an important approach for lead-compound discovery and has been successfully used in multiple projects [29, 30]. Recently, several crystal structures of CARM1 were determined, providing a prerequisite for structure-based virtual screening [26, 31–33]. Herein, due to the convenience and low cost of this approach, docking-based virtual screening was utilized to identify novel inhibitors of CARM1 from the Specs database (http://www.specs.net/). The candidates selected by virtual screening were then tested by biochemical experiments and eventually two novel inhibitors of CARM1 were identified. Among them, the more potent inhibitor DC_C66 displayed selectivity against PRMT1, PRMT6, and PRMT5. Molecular docking was conducted to investigate the binding modes of these inhibitors and molecular basis of selectivity for CARM1. Furthermore, cellular studies revealed that both inhibitors exhibited antiproliferation activity in several CARM1-associated cancer cell lines. Overall, this study has provided chemical probes in exploring biological functions of CARM1 and information for further optimization of potent inhibitors.

## 2. Materials and Methods

### 2.1. Virtual Screening Protocol

#### 2.1.1. Protein Preparation

The crystal structure of CARM1 in complex with indole inhibitor (PDB code 2Y1W) was used as a target for subsequent virtual screening [26]. The water molecules and ions were initially removed. The protein status was optimized through the Protein Preparation Wizard Workflow provided in the Maestro [34], with a pH value of 7.0 ± 2.0. Other parameters were set as the default. Residues within a distance of 6 Å around indole inhibitor were defined as binding pocket.

#### 2.1.2. Ligand Database Preparation

The Specs database (http://www.specs.net/), containing ~287,000 compounds, was utilized for the virtual screening. To refine the database, we filtered it by Lipinski's rule of five [35] and removed pan-assay interference compounds (PAINS) [36–38] with Pipeline Pilot, version 7.5 (Accelrys Inc., San Diego, CA, USA) [39], yielding a database of around 180,000 small-molecule compounds. The remaining molecules were treated by LigPrep [40] to generate all stereo isomers and different protonation states with Epik.

#### 2.1.3. Virtual Screening Protocol

**Figure 1:**
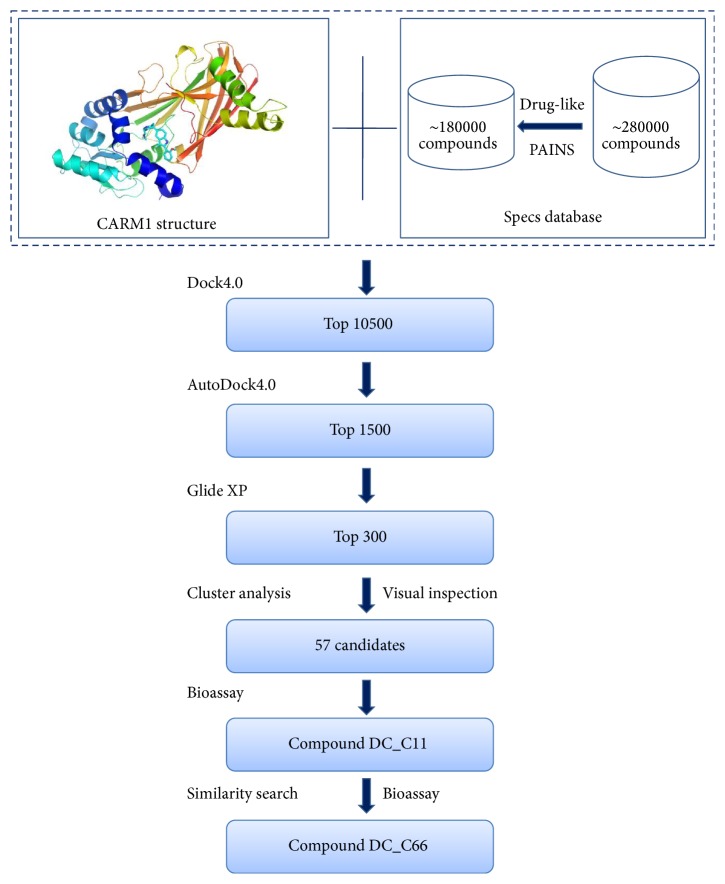
Flowchart of virtual screening procedures for CARM1 inhibitors.

The virtual screening protocol is shown in Figure 1. Firstly, the energy scoring function of DOCK4.0 was used to dock the compound library into the defined binding site. The top-ranked 10500 candidates selected by DOCK4.0 were further evaluated and ranked by the AutoDock4.0 program, leading to a list of 1500 compounds. The program Glide 5.5 [41] in XP mode [42] was run to calculate the free binding energy between these 1500 compounds and CARM1 protein. In order to ensure diversity in the candidates, the top 300 compounds from Glide 5.5 were classified to 30 groups by SciTegic functional class fingerprints (FCFP_4) in Pipeline Pilot, version 7.5 (Accelrys Inc., San Diego, CA, USA) [39], and 1–3 compounds were picked from each group. Finally, 57 compounds were selected and purchased for biological evaluation.

### 2.2. Similarity-Based Analog Searching

According to the results of the biological tests, we used the compound DC_C11 to run a two-dimensional similarity search through the prepared Specs database using Similarity Filter from File in Pipeline Pilot, version 7.5 (Accelrys Inc., San Diego, CA, USA). We purchased 10 compounds and tested their biological activity towards CARM1.

### 2.3. *In Vitro *CARM1 Enzyme Inhibition and Selectivity Assay

The enzymatic inhibitory activities of compounds were measured by the AlphaLISA assay provided by Shanghai ChemPartner Co., Ltd. The compounds selected from virtual screening were transferred to the assay plate (white opaque OptiPlate-384, PerkinElmer). 5 *μ*L of enzyme solution (final concentration was 0.1 nM) or pH 8.0 tris-based assay buffer (for Min well) was added to the assay plate and then centrifuged at 1000 rpm for 1 min. Afterwards, the assay plate was incubated for 15 min at room temperature (RT). Then 5 *μ*L of biotinylated H3 peptide/SAM mix (final concentrations were 50 nM and 300 nM, resp.) was added to the assay plate, which was covered with TopSeal-Afilm and incubated for 1 h at RT after centrifuging at 1000 rpm for 1 min (DMSO final concentration 1%). Next, 5 *μ*L of acceptor beads (final concentration was 10 *μ*g/mL) was added to stop the enzymatic reaction. After incubating at room temperature for 60 min, 10 *μ*L of donor beads was added (final concentration was 10 *μ*g/mL) in subdued light and then centrifuged at 1000 rpm for 1 min. Finally, the mixtures were incubated for 30 min at RT, and the signal was read in alpha mode using EnVision readers. The IC_50_ values were calculated by fit inhibition rates under different concentrations into GraphPad Prism 5.0 software.

### 2.4. Cell Viability Assay

The three cell lines, HELA, K562, and MCF7, were purchased from the American Type Culture Collection (ATCC). HELA, K562, and MCF7 were cultured in DMEM (Life Technologies) supplemented with 10% FBS. All of the cell lines were seeded into 96-well plates at an appropriate density and then treated with compounds of different concentrations or DMSO control. After 24 hrs, 48 hrs, and 72 hrs, cell viabilities were measured by the MTT assay.

### 2.5. Binding Energy Calculations

In order to investigate the binding mode of DC_C11 and DC_C66, molecular docking was performed using Glide 5.5 in XP mode. The generated conformations were then used for binding energy calculations by Prime MM-GBSA (Molecular Mechanics/Generalized Born Surface Area method) [43]. The binding energy was calculated as follows:

(1) ΔG=E_complexminimized−E_ligandminimized+E_receptor.

In the calculations, the protein flexibility was set to 12 Å.

## 3. Results and Discussion

### 3.1. Structure-Based Virtual Screening

In this study, docking-based virtual screening was performed to identify CARM1 inhibitors with novel scaffolds, and the flowchart is shown in Figure 1. The crystal structure of CARM1 in complex with indole inhibitor (PDB code 2Y1W) was used as a target for the following* in silico* screening [26]. Residues within a distance of 6 Å around indole inhibitor were defined as binding pocket, which contains the binding site of AdoMet and the arginine substrate. The Specs database (http://www.specs.net/), containing ~287,000 compounds, was utilized for the virtual screening. To refine the database, we filtered it by Lipinski's rule of five and removed pan-assay interference compounds (PAINS) [36–38] with Pipeline Pilot, version 7.5 (Accelrys Inc., San Diego, CA, USA) [39], yielding a database of around 180,000 small-molecule compounds, which were subsequently docked and ranked with different score functions. The top-ranked 10500 candidates selected using energy scoring function of DOCK4.0 [44] were subsequently evaluated and ranked by the AutoDock4.0 program [45], yielding a list of 1500 compounds. Then, the program Glide 5.5 (XP mode) [42] was chosen to calculate the free energy of binding between these 1500 compounds and CARM1 protein. According to the docking scores, the top-ranked 300 were clustered using Pipeline Pilot to ensure the scaffold diversity in the primary hits. The clustered molecules were cherry-picked by visual inspection based on the following considerations. (1) At least one compound was selected in each clustered group. (2) The binding modes were reasonable and molecules not occupying the SAM or substrate binding pocket were not chosen. (3) Among a group of similar molecules, compounds with lower molecular weight were preferred. Finally, 57 compounds were purchased for further biochemical validation.

### 3.2. Enzyme Inhibition and Selectivity Assay

All of the selected 57 candidate molecules were tested for CARM1 inhibition to determine their biochemical activities. Here, AlphaLISA assay, which is a powerful and versatile platform, was performed to test the inhibitory activities of the compounds. The enzyme solution and compounds or assay buffer were transferred to assay plates, which was incubated at RT. Then 5 *μ*L of biotinylated H3 peptide/SAM mix was added and incubated for 1 h at RT. Afterwards, acceptor and donor beads were added sequentially. The end point was read in alpha mode using EnVision readers, and IC_50_ values were calculated in GraphPad Prism 5.0 software. Among these candidates, only one compound DC_C11 was found to be active for CARM1 inhibition, which showed an IC_50_ value of 15 *μ*M (Table 1). We used this core structure as a hit to perform a two-dimensional similarity search through the Specs database by Pipeline Pilot, version 7.5 (Accelrys Inc., San Diego, CA, USA) [39], leading to a compound DC_C66 which displayed inhibitory better potency for CARM1 with IC_50_ values of 1.8 *μ*M.

To investigate the selectivity of the compounds, we tested the inhibitory activities of compounds DC_C11 and DC_C66 against several selected members of type I PRMT family, including PRMT1 and PRMT6 (Table 1). It was seen that DC_C66 showed relatively weaker activity against PRMT1 and PRMT6. Moreover, DC_C66 also showed little inhibitory activity of PRMT5, a member of type II PRMT, by <50% inhibition rate at a concentration of 50 *μ*M. Taken together, these results indicated that DC_C66 has a good selectivity for CARM1 against other selected PRMTs.

### 3.3. Cell-Based Activity

**Figure 2:**
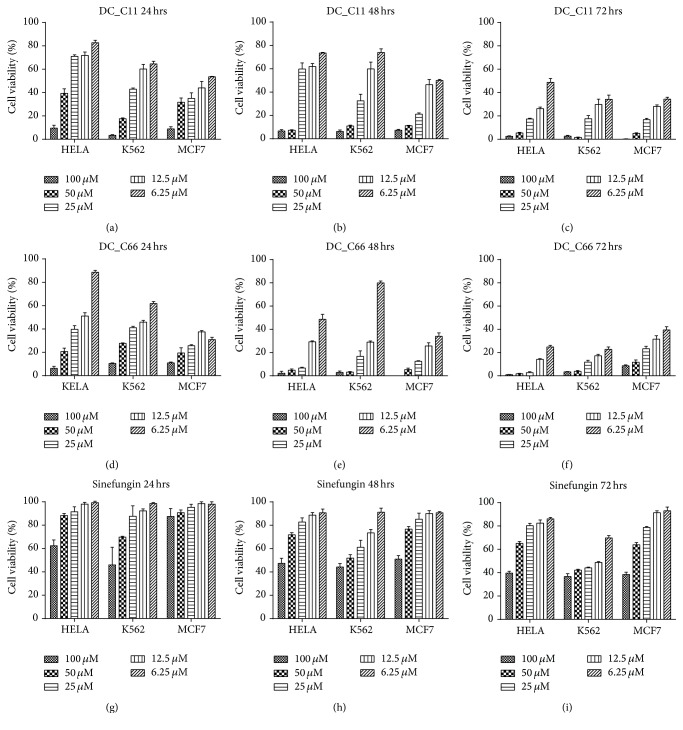
Antiproliferative effect of DC_C11 and DC_C66 on several cancer cell lines. (a–c) Time-dependent and dose-dependent inhibitory effect of DC_C11 on HELA, K562, and MCF7 within 24 hrs, 48 hrs, and 72 hrs, respectively. (d–f) Time-dependent and dose-dependent inhibitory effect of DC_C66 on HELA, K562, and MCF7 within 24 hrs, 48 hrs, and 72 hrs, respectively. (g–i) Time-dependent and dose-dependent inhibitory effect of Sinefungin on HELA, K562, and MCF7 within 24 hrs, 48 hrs, and 72 hrs, respectively.

It has been reported that CARM1 was a potential target in many cancers; thus it is well accepted that inhibiting CARM1 could affect cancer cell proliferation. In this study, three human tumor cell lines including HELA (cervical cancer), K562 (myeloid leukemia), and MCF7 (breast cancer) were chosen to evaluate the cellular activity of the two compounds DC_C11 and DC_C66* in vivo*. Sinefungin, a pan-PRMTs inhibitor which has the same scaffold as the cofactor SAM does, was evaluated for control experiment [46]. As shown in Figure 2, both DC_C11 and DC_C66 could inhibit proliferation of cancer cells in a time-dependent and dose-dependent manner while Sinefungin presented weaker inhibitory activity in cellular level. In the three cell lines, DC_C66 presents better antiproliferative cellular activity, which is consistent with their inhibitory activity* in vitro. *Combined with the biological data* in vitro*, we confirmed that compounds DC_C11 and DC_C66 are cell membrane permeable, which presented promising activity both* in vitro* and in cellular environment.

### 3.4. Binding-Mode Analysis

**Figure 3:**
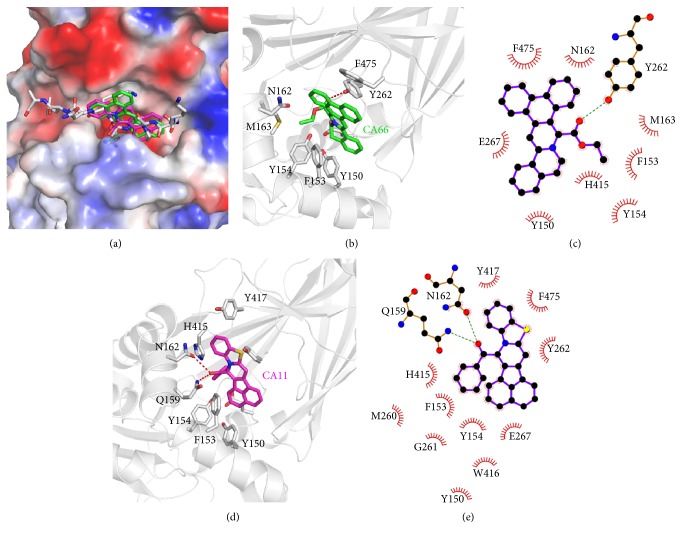
Predicted binding mode of DC_C11 and DC_C66 with CARM1 from docking analysis. (a) Superimposition of the binding modes of the two compounds and substrate H3 peptide (PDB ID: 5DX0). The structure of CARM1 is displayed in vacuum electrostatics. H3 peptide is shown as gray sticks, DC_C11 is shown as magenta sticks, and DC_C66 is displayed as green sticks. (b) A close view of the interactions between DC_C66 and CARM1 in the binding pocket; the key residues are shown as sticks. (c) Schematic diagram showing putative interactions between CARM1 and DC_C66. Residues involved in the hydrophobic interactions are shown as starbursts, and hydrogen-bonding interactions are denoted by dotted green lines. (d) A close view of the interactions between DC_C11 and CARM1 in the binding pocket; the key residues are shown as sticks. (e) Schematic diagram showing putative interactions between CARM1 and DC_C11.

To further understand the possible binding mode of DC_C11 and DC_C66 with CARM1, molecular docking study was performed with Glide in XP mode. As shown in Figure 3(a), both of DC_C11 and DC_C66 fit into the negative-charged binding pocket of substrate arginine in H3 peptide [33], implying that the compounds inhibit the activity of CARM1 by destroying the protein-protein interactions between CARM1 and substrate peptide. The phenyl ring bulks of DC_C11 and DC_C66 establish hydrophobic interactions with Y150, F153, Y154, N162, M163, and F475 in active site; the majority of these residues participate in interactions between CARM1 and its substrates (Figure 3) [33]. Besides, DC_C66 forms hydrogen bond with Y262 which probably accounts for its ability to inhibit CARM1 activity (Figures 3(b) and 3(c)). Polar interactions between the oxygen in the carbonyl group of DC_C11 and side chain of Q159 as well as N162 also occur (Figures 3(d) and 3(e)). We further calculate binding energies of two compounds using Prime MM-GBSA [47] (Table 2). The results showed that DC_C11 binds to the substrate binding pocket with lower binding energy (−26.72 kcal/mol), followed by DC_C66 with a higher value (−34.71 kcal/mol). The calculated binding energies are in accordance with that of activity, rationalizing our experimental data of bioassays.

The sequence alignment and structural superposition of CARM1, PRMT1, and PRMT6 reveal several differences between these proteins (Figures S2 A-B), which may contribute to selectivity of the CARM1 inhibitors. In the N-terminal helix, which is disordered in the crystal structure of rat PRMT1 and is essential for the enzymatic activity [48], the corresponding residues of F153 in CARM1 are S39 in PRMT1 and C50 in PRMT6 (Figures S2 A-B). Besides, F475 in C-terminal of CARM1 corresponds to R353 in PRMT1 and E374 in PRMT6. Since F153 and F475 are important components of the hydrophobic pocket that accommodates the phenyl ring bulk of DC_C66 (Figure 3), substitutions of the phenylalanine with hydrophilic amino acids may decrease the binding affinity of the CARM1 inhibitors (Figures S2 A-B). These comparisons theoretically explain the selectivity of DC_C66 against CARM1 from the molecular basis.

## 4. Conclusion

Posttranslational modifications of proteins have been increasingly recognized as essential modulators to their function in cells. In particular, arginine methylation, an important posttranslational modification, is catalyzed by PRMTs. CARM1, a member of PRMTs, has been implicated in a variety of cancers. Thus, the identification of selective inhibitors of CARM1 as probes to investigate CARM1 cellular function and its relevance in disease would be of significant interest in the field of epigenetics. Here in our study, by combining structure-based virtual screening and biochemical assays, we have identified DC_C11 and DC_C66 as novel inhibitors of CARM1, with IC_50_ values of 15 and 1.8 *μ*M, respectively. Notably, DC_C66 displayed good selectivity against PRMT1, PRMT6, and PRMT5. The binding-mode prediction revealed that the two compounds can efficiently bind in the substrate binding site of CARM1 and thus inhibit the enzymatic activity by destruction of protein-protein interactions between CARM1 and its various substrates. Furthermore, the two compounds showed good cell permeability and blocked the proliferation of several cancer cells related to CARM1 overexpression. Overall, this study demonstrated an efficient docking-based virtual screening procedure that can be used to identify novel CARM1 inhibitors. These results paves the way for further development of inhibitors with novel scaffolds and functional probes to target CARM1 on the cellular level for both biological and therapeutic purposes.

## Supplementary Material

The Supplementary Material contains Figure S1 which displays The chemical structures of typical reported inhibitors targeting CARM1, and Figure S2 which describes the molecular basis for selectivity of compound DC_C11 and DC_C66.

## Figures and Tables

**Table 1:** Chemical structures and inhibitory activity (^a^IC_50_, *μ*M) of selected compounds based on virtual screening against CARM1 and several other PRMTs.

Compound ID	Specs ID	Compound structure	IC50 (*μ*M)		
			CARM1	PRMT1	PRMT6
DC_C66	AQ-405/42300312	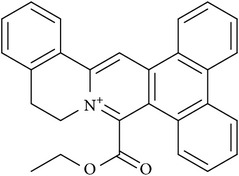	1.8	21	47
DC_C11	AQ-405/42300392	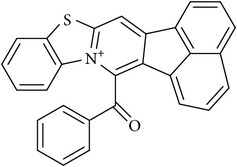	15	36	41

^a^All assays were conducted in duplicate.

**Table 2:** Binding energy for compounds DC_C66 and DC_C11.

Compound ID	DC_C66	DC_C11

Binding energy (kcal/mol)	−34.71	−26.72
