# Supplementary material for: Identification of Novel Inhibitors against Coactivator Associated Arginine Methyltransferase 1 Based on Virtual Screening and Biological Assays
Source: Biomed Res Int. 2016 Oct 27;2016:7086390. doi: 10.1155/2016/7086390 (PMC5107250; doi:10.1155/2016/7086390)
Supplement: Supplementary file 1 — The Supplementary Material contains Figure S1 which displays The chemical structures of typical reported inhibitors targeting CARM1, and Figure S2 which describes the molecular basis for selectivity of compound DC_C11 and DC_C66. [file 7086390.f1.pdf]

Identification of Novel Inhibitors against Co-activator Associated Arginine Methyltransferase 1 (CARM1) Based on Virtual Screening and Biological Assays

Fei Ye[a,b,†,*], Weiyao Zhang[a,†], Wenchao Lu[c,d], Yiqian Xie[c], Hao Jiang[c,d], Jia Jin[a, *], Cheng Luo[c]

[a]Zhejiang Sci-Tech University, College of Life Sciences, Hangzhou, China

[b]Key Laboratory of Plant Secondary Metabolism and Regulation of Zhejiang Province

[c]Drug Discovery and Design Center, State Key Laboratory of Drug Research, Shanghai Institute of Materia Medica, Chinese Academy of Sciences, China

[d]University of Chinese Academy of Sciences, Beijing, China

[*]Corresponding author:

Tel: +86 571 86843811; E-mail address: yefei@zstu.edu.cn (Fei Ye).

Tel: +86 571 86843199; E-mail address: aukaukauk@163.com (Jia Jin).

†These authors contributed equally

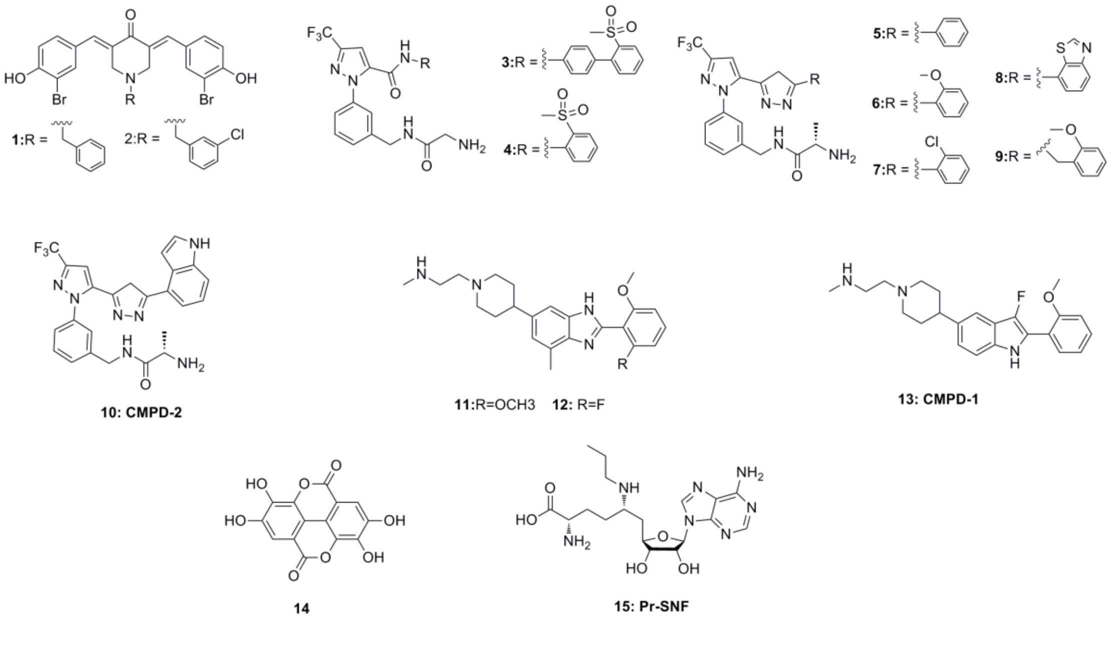

Figure S1. The chemical structures of typical reported inhibitors targeting CARM1.

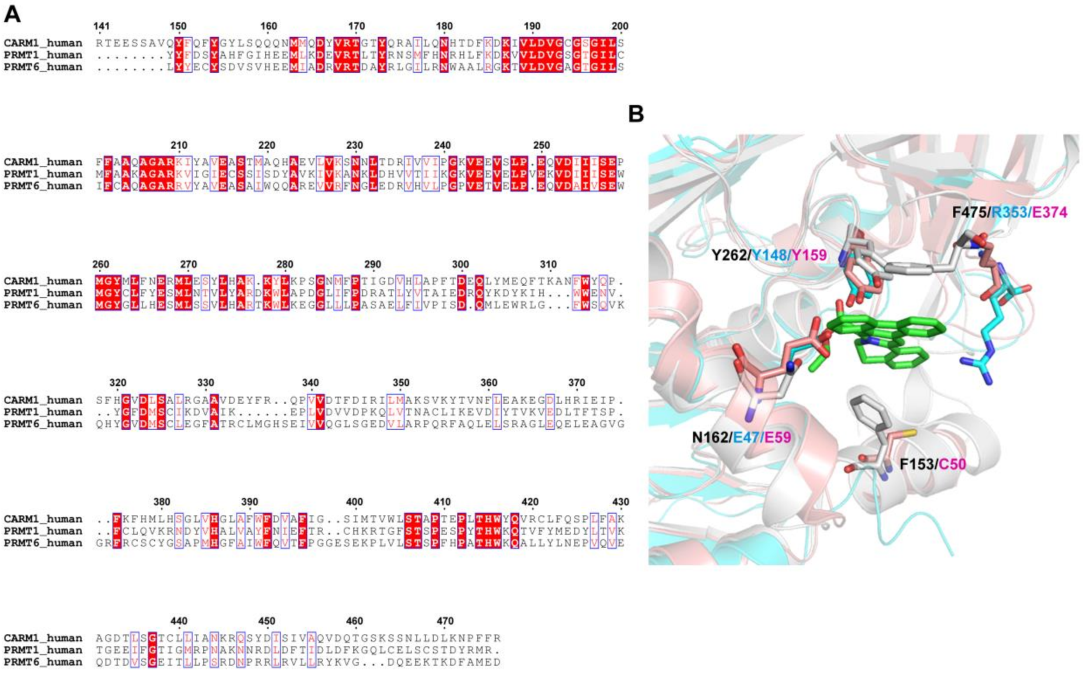

Figure S2. The molecular basis for selectivity of compound CA_C11 and CA_C11. (A) Sequence alignment of CARM1 (human), PRMT1 (human), and PRMT6 (human). (B) Structural superimposition of active sites of CARM1, PRMT1 and PRMT6. CARM1 (PDB ID code: 2Y1W), PRMT1 (IORH) and PRMT6 (5E8R) are colored grey, cyan and pink, respectively.
